# Clinical Evidence and Risk Factors for Reproductive Disorders Caused by Bacterial Infections in Meat Goats in Northeastern Thailand

**DOI:** 10.1155/2022/1877317

**Published:** 2022-02-08

**Authors:** Sarinya Rerkyusuke, Sawarin Lerk-u-suke, Anucha Sirimalaisuwan

**Affiliations:** ^1^Division of Livestock Medicine, Faculty of Veterinary Medicine, Khon Kaen University, Khon Kaen 40002, Thailand; ^2^Research Group of Animal Health Technology, Faculty of Veterinary Medicine, Khon Kaen University, Khon Kaen 40002, Thailand; ^3^Department of Geographic Information Science, School of Information and Communication Technology, University of Phayao, Phayao 56000, Thailand; ^4^Research Unit of Spatial Innovation Development, School of Information and Communication Technology, University of Phayao, Phayao 56000, Thailand; ^5^Department of Veterinary Bioscience and Veterinary Public Health, Faculty of Veterinary Medicine, Chiang Mai University, Chiang Mai 50100, Thailand

## Abstract

The objective of this study was to identify risk factors related to reproductive disorders caused by bacterial infections in goats in northeastern Thailand. Two hundred twenty farms were investigated, and 49 herds were found to have clinical reproductive disorders. Moreover, 96% (47/49) of herds showing clinical reproductive failure preferred to circulate bucks between herds. A total of 118 sera, including 85 clinical reproductive disorder cases such as abortion (*n* = 70), abortion with arthritis (*n* = 1), orchitis (*n* = 3), repeat breeder (*n* = 6), sterile (*n* = 1), and weak kids (*n* = 4), and 33 bucks' circulations were serologically tested for bacterial infections caused by *Coxiella burnetii*, *Chlamydophila abortus*, and *Brucella* spp. Results showed 69% (81/118 cases) were seropositive for Q fever (*n* = 55; 46.61%), brucellosis (*n* = 8; 6.78), and chlamydiosis (*n* = 18; 15.25%), respectively; 82% of herds (40/49 herds) were infected with at least one of those diseases. Moreover, 40% of infected herds (16/40) had coinfection among the three of those diseases. Approximately 60% (20/33) of buck circulation showed seropositivity to at least one of the diseases, and 85% of infected bucks were seropositive for Q fever (17/20). Buck circulation between herds is a risk factor for diseases on farms (*p*=0.001); odds ratio (OR = 109.29; 95% confidence interval (CI) = 6.61–1,807.38). Moreover, the annual brucellosis test is a protective factor against reproductive failure cases on farms (*p*=0.022; OR = 0.45; 95% CI = 0.23–0.89). Reproductive disorder cases can be caused by sexual transmission, so buck circulation can yield Q fever, brucellosis, and chlamydiosis in communities. This investigation is the first report of chlamydiosis infection in our area. Concerning Q fever, chlamydiosis, and brucellosis are zoonotic diseases that impact animal health and production losses. Control and prevention measures related to risk factors together with active surveillance programs should be incorporated into client education.

## 1. Introduction

Bacterial infection-induced reproductive disorders, such as brucellosis, Q fever, enzootic abortion (chlamydiosis), caseous lymphadenitis (CLA), listeriosis, campylobacteriosis, leptospirosis, salmonellosis can be found in Caprinae [1].

Q fever is caused by Gram-negative bacteria called *Coxiella burnetii*. As for clinical signs, does and nannies show abortions in the first trisemester, stillbirths, a retained placenta, endometritis, infertility, weak kids, and repeat breeding [2]. Chlamydiosis is caused by Gram-negative bacteria called *Chlamydophila abortus*. Infected doe goats show signs of reproductive failure, including abortion, miscarriage, stillbirths, and weak kids. In addition, orchitis, epididymis, and seminal vesiculitis can occur in bucks. Caprine brucellosis is caused by Gram-negative coccobacilli bacteria named *Brucella melitensis*. Regarding clinical signs, pregnant goats show placentitis and abortion in the second to third semesters or premature births, weak kids, a retained placenta, mastitis, metritis, repeat breeding, or infertility. Infected bucks may show signs of orchitis or epididymitis [1, 3]. Other clinical signs may be sterility and arthritis [3].

Infected herds face economic losses due to the clinical signs of infection, such as abortion, infertility, still births, miscarriage in does, and sterile orchitis in bucks, which can reduce productivity. Moreover, zoonotic diseases can be transmitted to humans.

Information on bacterial infection causing reproductive failure in caprine was limited in northeast Thailand. The aim of this study is to determine the presence of antibodies against Q fever, chlamydiosis, and brucellosis, which can cause reproductive disorders in goats. Additionally, the risk factors associated with seropositivity in these infectious diseases are necessary for prevention and control measures due to their being zoonotic diseases.

## 2. Materials and Methods

### 2.1. Study Area

A retrospective study was conducted on the Caprine Brucellosis Surveillance Program being conducted by the Academic Service of the Faculty of Veterinary Medicine, Khon Kaen University, from March 2017 to September 2020. In total, 220 smallholder goat herds from 11 provinces including Buriram (4), Chaiyaphum (97), Khon Kaen (59), Kalasin (2), Mahasarakham (13), Nongbualamphu (5), Nakhon Ratchasima (5), Roi Et (7), Sakon Nakhon (21), Srisakate (3), and Udon Thani (4) were included in services.

### 2.2. Sampling

Serum samples were collected from every goat aged over 4 months based on the criteria of the Brucellosis Surveillance Program [4, 5]. In total, 4,810 goats in 220 herds were tested for brucellosis by serological testing conducted by the Academic Service of the Faculty of Veterinary Medicine, Khon Kaen University.

Twenty-two percent (49/220) of smallholder goat herds were found to have clinical reproductive disorders in at least one case on farms in 8 provinces. A total of 118 were found to have reproductive disorders, such as abortion (*n* = 70), abortion with arthritis (*n* = 1), orchitis (*n* = 3), repeat breeding (*n* = 6), sterility (*n* = 1), and weak kids (*n* = 4), and bucks' circulation between herds (*n* = 33) was included in serological testing for Q fever and chlamydiosis.

### 2.3. Serological Tests

Blood samples were collected from the jugular vein into 5 ml Vacutainer® red tubes. All samples were transported on ice to the laboratory at the Faculty of Veterinary Medicine, Khon Kaen University, within 6 h. Afterwards, all samples were centrifuged at 2,500 rpm for 10 min for serum collection and then stored at −20°C until analysis.

Screening for brucellosis was performed following the OIE standard procedure of the Rose Bengal technique, and the positive samples were confirmed by the complement fixation test [5].

IgG antibodies against *C. burnetii* and *C. abortus* infection were detected by the indirect ELISA IDEXX Q Fever Ab test and IDEXX chlamydiosis Total Ab test (IDEXX Switzerland AG, Stationsstrasse 12, CH-3097 Liebefeld-Bern, Switzerland). The procedures were carried out according to the instructions of the manufacturer. The optical densities (OD) of the samples and controls were measured at 450 nm using a spectrophotometer. The S/P% was determined based on the OD following the equation:

(1) $$\begin{array}{c} \frac{S}{P\%}=100\times \frac{\text{Sample }A\left(450\right)-\text{NC}\bar{x}\;A\left(450\right)}{\text{PC}\bar{x}-\text{NC}\bar{x}}. \end{array}$$

The results were classified according to the manufacturer's instructions as follows.

IDEXX ELISA; S/P% > 40, 30 < S/P% < 40, and S/P% <30 were positive, suspect, and negative samples, respectively. The sensitivities of the test were 100% and 89–95% for the Q fever and chlamydiosis tests, respectively. The specificity of the test was 100% for both tests [6].

### 2.4. Questionnaires

One hundred eighty-nine owners were interviewed for their management of risk factors associated with the presence of disease. Factors included herd structure and herd and health management especially, history of buck in herd, movement and quarantine, personnel hygiene for feeding animals, barn cleaning, and giving birth to animals during parturition. Additionally, the herd's annual brucellosis testing was included in the questionnaires.

### 2.5. Data Analysis

#### 2.5.1. Statistical Analysis

Data were analysed using Microsoft Office Excel 2016 and MedCalc® version 19.8 (MedCalc Software Ltd., 2021). The univariate analysis of risk factors was performed to identify variables associated with the positive herds at a significance level of 0.05 as the random effect in the model.

#### 2.5.2. Epidemiological Analysis

The spatial distribution was analysed using Quantum GIS. The geographic coordinate system was used to produce the map for epidemiologic analysis.

## 3. Results and Discussions

### 3.1. Clinical Evidence Associated with Seropositivity and Spatial Distribution of Q Fever, Chlamydiosis, and Brucellosis in Meat Goat Herds

Based on clinical evidence of reproductive disorders in this area, 81.63% (40/49) of the investigated herds already had bacterial infection due to seropositivity against Q fever, chlamydiosis, or brucellosis infection. Only 9 herds (9/49; 18.36%) did not show antibodies against these bacterial infections by serological testing. Seropositivity to Q fever, chlamydiosis, and brucellosis testing in the herd was 45.0% (*n* = 18/40), 25.0% (*n* = 10/40), and 30.0% (*n* = 12/40), respectively. In addition, 16 herds (40.00%) had coinfection for at least two of three diseases.

At the individual case level, seropositivity to Q fever, chlamydiosis, and brucellosis infection were 46.61% (55/118), 15.25% (18/118), and 6.78% (8/118), respectively. Abortion (71/118; 60.16%) was the most common reproductive disorder in this area. Other clinical signs, such as orchitis, repeat breeding, sterility, and weak children were found in less than 15%. Interestingly, 41 herds shared 33 circulations of bucks with other herds, and 60.6% of bucks (20/33) were seropositive with at least one of these infections. Eighty-five percent of infected bucks were seropositive for Q fever (17/20), followed by chlamydiosis in approximately 15% (3/20) and brucellosis at 5% (1/20). Seropositivity for brucellosis was the lowest in proportion in this area in comparison to Q fever and chlamydiosis. This might have been due to the active surveillance program for brucellosis, which screens for brucellosis annually in registered goat herds [7]. However, a few cases of reproductive failure caused by *Brucella* spp. still occurred in northeastern Thailand.

Clinical reproductive disorders and bucks' circulation related to serological testing are shown in Table 1.

### 3.2. Risk Factors

Herd and health management factors related to Q fever, chlamydiosis, and brucellosis infection were determined based on interviews with 189 owners of the 47 infected herds and 142 noninfected herds. All herds used natural mating for breeding. Buck circulation between herds was a highly significant risk factor for seropositivity to at least one of those diseases in herds (*p*=0.001; odds ratio (OR) = 109.29; 95% confidence interval (CI) = 6.61–1,807.38). In addition, brucellosis testing annually was a highly significant protective factor for reproductive failure cases on farms (*p*=0.022; OR = 0.45; 95% CI = 0.23–0.89; Table 2). Although other factors were not significantly associated with clinical reproductive disorders in this study, brucellosis testing before movement and quarantine at least 30 days before introduction to herds could reduce the opportunity for the introduction of pathogens from outside to the herds. Additionally, separating birth places from other areas and personnel hygiene during care of pregnant herds could protect farmers from pathogenic transmission while working on the farm.

### 3.3. Spatial Distribution of Q Fever, Chlamydiosis, and Brucellosis in Smallholder Goat Herds

The presence of reproductive disorder on farms related to bucks' circulation between herds was as follows: geographic information system data (GIS) showed that 93.93% of bucks (31/33) were seropositive to at least one Q fever, chlamydiosis, or brucellosis infection among the 41 herds.

**Figure 1:**
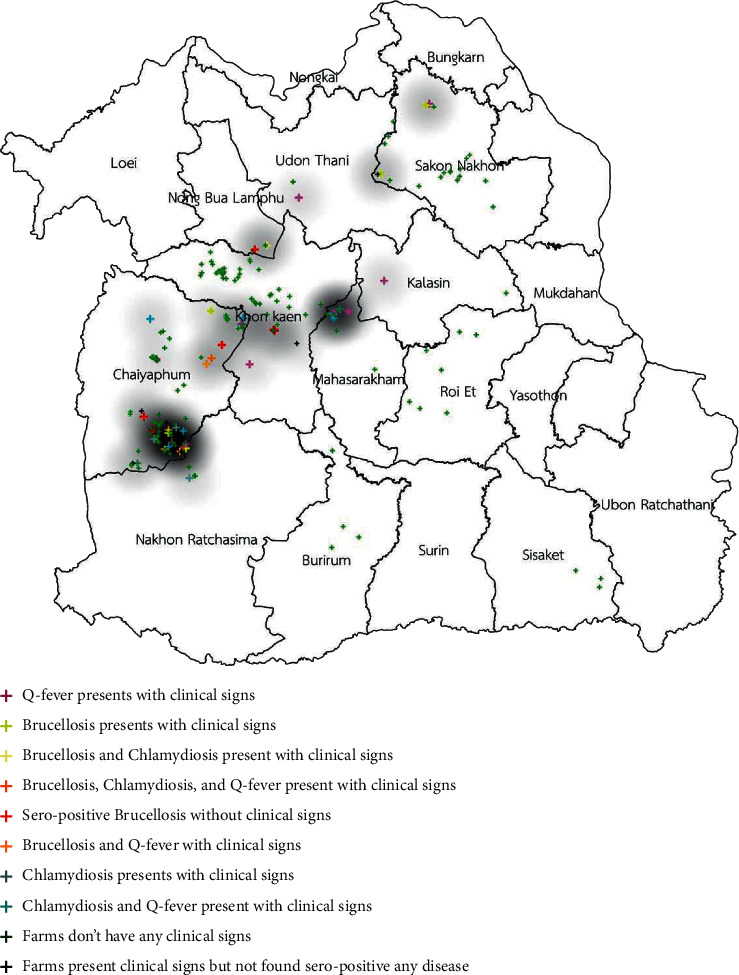
Distribution of clinical cases of brucellosis, chlamydiosis, and Q fever infection in smallholder meat goat herds in northeastern Thailand.

**Figure 2:**
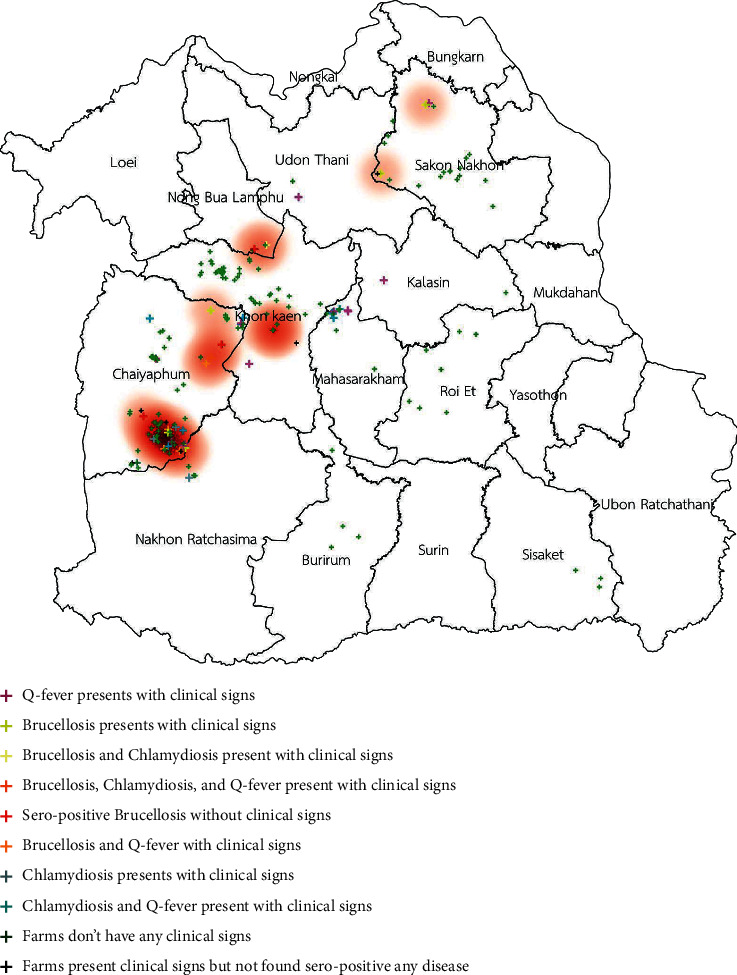
Distribution of clinical cases of brucellosis infection in smallholder meat goat herds in northeastern Thailand.

**Figure 3:**
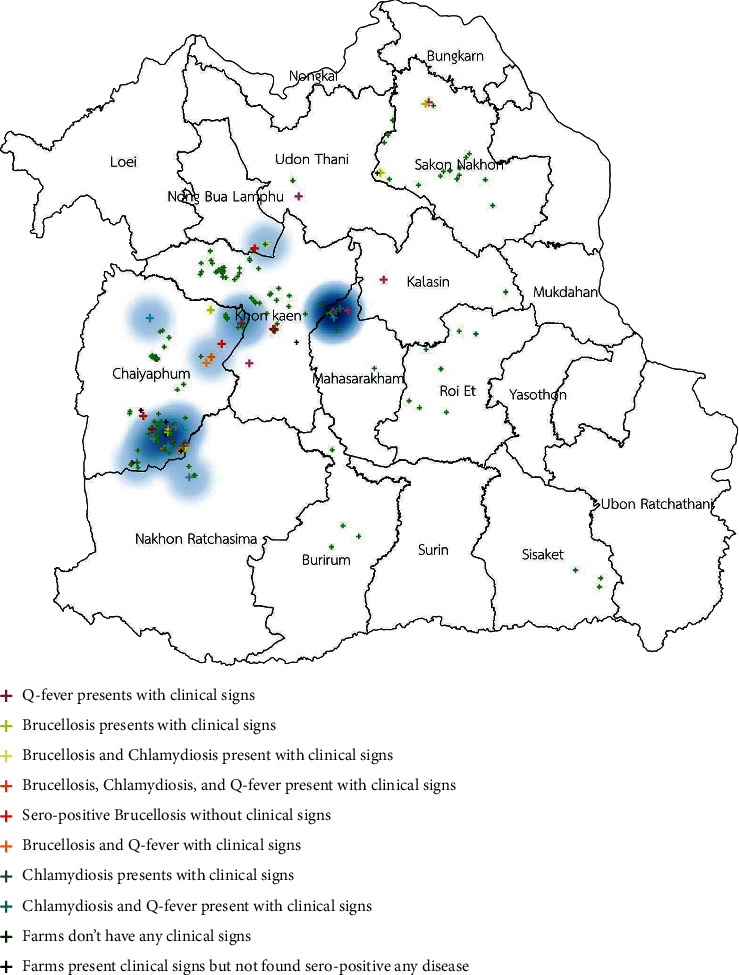
Distribution of clinical cases of chlamydiosis infection in smallholder meat goat herds in northeastern Thailand.

**Figure 4:**
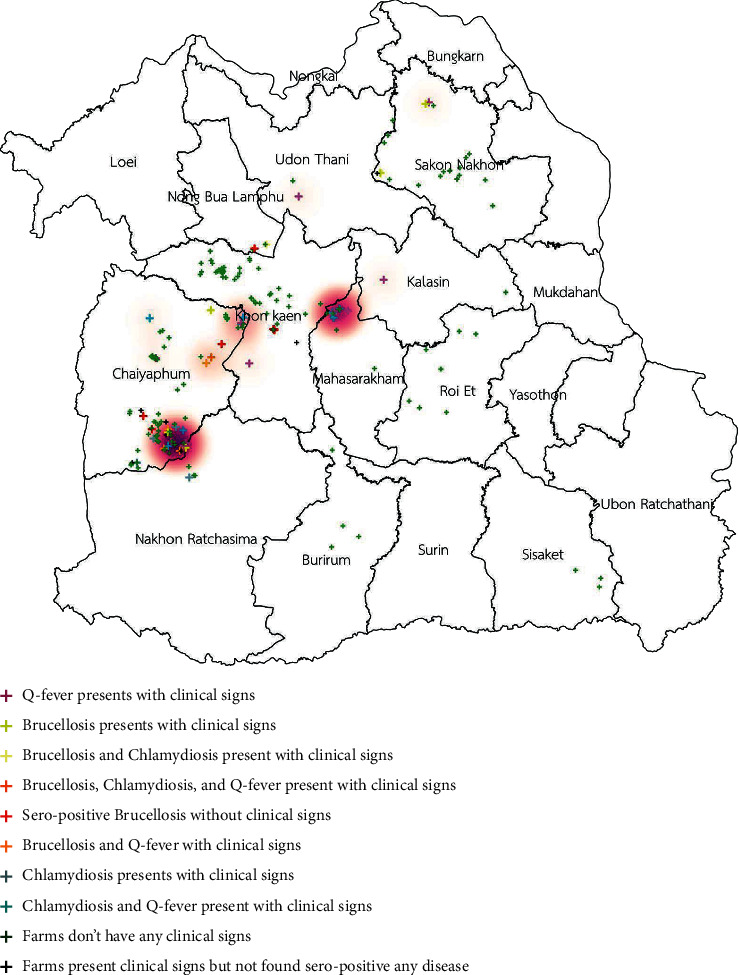
Distribution of clinical cases of Q fever in smallholder meat goat herds in northeastern Thailand.

**Figure 5:**
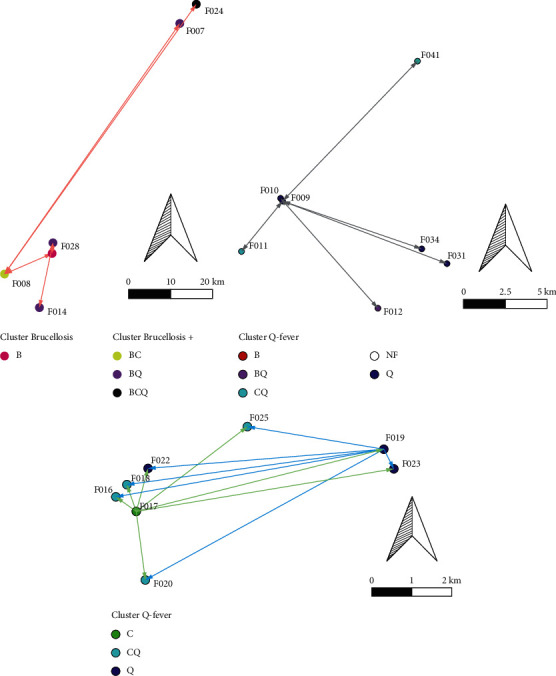
Cluster surge of brucellosis, Q fever, and chlamydiosis across herds within three communities due to shared buck circulation.

The spatial distribution of three of those diseases is shown in Figure 1. The distribution of clinical cases of brucellosis, chlamydiosis, and Q fever is shown in Figures 2–4. The interviews showed that farmers preferred to use natural mating to breed animals in their herds, and bucks' circulation between herds was commonly found in their communities. The GIS showed the cluster of Q fever-, chlamydiosis-, and brucellosis-infected herds that circulated the bucks in their community from one main herd to another (Figure 5). Frequently, bucks' circulation between herds not only spreads pathogens via natural mating with does, but pathogens can also spread to other healthy animals by inhalation, ingestion, and direct contact with contaminated material, such as faeces and urine of infected bucks. Most infected herds were coinfected with those diseases, and these might cause severe cases of reproductive disorders in goats.

Based on this investigation, the significant factor for controlling and preventing those diseases is avoiding bucks' circulation between herds. Although the serological screening of brucellosis is the only test in the Thai national brucellosis surveillance system, annual serological brucellosis testing was found to be a significant protective factor against bacterial reproductive disorders infection in herds. Additional advice for introducing animals, especially bucks, should be screening not only for brucellosis but also for Q fever and chlamydiosis.

Previous studies showed clinical cases of brucellosis and Q fever in humans related to livestock animals in Thailand [8–11]. Q fever, chlamydiosis, and brucellosis from small ruminants have been linked to zoonotic diseases between animals and humans and have caused serious human health problems worldwide [12–14]. In addition, these infections reduce the efficacy of animal health and production, decrease socioeconomic benefits, and increase the costs of human health care and communities resulting from the need for laboratory diagnosis, treatment, and control [15–20].

In northeastern Thailand, reproductive disorders have been reported to be caused by other diseases, such as epididymo-orchitis caused by *Burkholderia pseudomallei* [21], orchitis and mastitis caused by *Corynebacterium pseudotuberculosis* [22], abortion, still birth, and infertility with a low conception rate caused by *Leptospira noguchii* [23]. However, nine herds had reproductive failure and did not show antibodies against Q fever, chlamydiosis, brucellosis, melioidosis, caseous lymphadenitis, and leptospirosis. This might require further investigation for other bacterial infections, such as *Campylobacter* infection, listeriosis, and salmonellosis; other protozoa infections, such as neosporosis and toxoplasmosis; and virus infections such as *Pestivirus* infection.

## Figures and Tables

**Table 1:** Description of clinical reproductive disorders and bucks' circulation related to seropositivity for Q fever, chlamydiosis, and brucellosis in 49 smallholder goat herds in northeastern Thailand.

Reproductive disorder	Case (*n*)	Positive Q fever (%)	Positive chlamydiosis (%)	Positive brucellosis (%)
Abortion	70	30 (42.85)	14 (20.00)	6 (8.57)
Abortion with arthritis	1	1 (100.00)	0 (0)	0 (0)
Orchitis	3	2 (66.67)	0 (0)	0 (0)
Repeat breeder	6	4 (66.67)	0 (0)	1 (16.67)
Sterile	1	1 (100)	0 (0)	0 (0)
Weak kids	4	0 (0)	1 (25.00)	0 (0)
Buck circulation	33	17 (51.51)	3 (9.09)	1 (3.03)
Total	118	55 (46.61)	18 (15.25)	8 (6.78)

**Table 2:** Univariate analysis of risk factors for bacterial infection-caused reproductive failures in smallholder goats herds in northeastern Thailand.

Factor	Infected herd (*n* = 47)	Noninfected herd (*n* = 142)	OR	95% CI	*p* value
Group population (together)	47	130	9.1	0.53–156.71	0.1376
Buck circulation (use)	47	66	**109.29**	**6.61**–**1,807.38**	0.0010
Birth place (have)	20	74	0.68	0.35–1.32	0.2572
Brucellosis testing before movement (have)	10	43	0.62	0.28–1.36	0.2362
Movement (3 months) (have)	42	116	1.88	0.68–5.22	0.2241
Quarantine 30 d (have)	6	22	0.8	0.3–2.11	0.6488
Brucellosis testing annual (have)	17	79	**0.45**	**0.23**–**0.89**	**0.0222**
Personnel hygiene: mask (have)	5	18	0.82	0.29–2.35	0.7114
Personnel hygiene: glove birth (have)	30	95	0.87	0.44–1.74	0.6998
Personnel hygiene: handwashing (have)	5	31	0.43	0.16–1.17	0.0977

Bold shows that significant association was classified as *p* < 0.05. OR : odds ratio; CI : confidence interval.

## Data Availability

Anyone can access the data by clicking on this link below https://docs.google.com/spreadsheets/d/1JE4OKLG7RAzRSm1BS85l3nUri2rGRSnw/edit?usp=sharing∼∼∼∼∼∼∼∼∼^∼^∼^∼^∼∼∼∼∼∼∼∼∼∼∼amp;ouid=106310776112596212448∼∼∼∼∼∼∼∼∼^∼^∼^∼^∼∼∼∼∼∼∼∼∼∼∼amp;rtpof=true∼∼∼∼∼∼∼∼∼^∼^∼^∼^∼∼∼∼∼∼∼∼∼∼∼amp;sd=true.
